# The Ander's organ: a mechanism for anti-predator ultrasound in a relict orthopteran

**DOI:** 10.1242/jeb.237289

**Published:** 2021-01-28

**Authors:** Charlie Woodrow, Kevin A. Judge, Christian Pulver, Thorin Jonsson, Fernando Montealegre-Z

**Affiliations:** 1 University of Lincoln, School of Life Sciences, Joseph Banks Laboratories, Green Lane, Lincoln LN6 7DL, UK; 2 Department of Biological Sciences, MacEwan University, Edmonton, Alberta, Canada T5J 4S2; 3 Department of Neurobiology & Behaviour, Institute of Biology, Karl-Franzens-University Graz, Universitätsplatz 2, 8010 Graz, Austria

**Keywords:** Grig, Insect, Bioacoustics, Predation, Secondary defences, Ancient

## Abstract

The use of acoustics in predator evasion is a widely reported phenomenon amongst invertebrate taxa, but the study of ultrasonic anti-predator acoustics is often limited to the prey of bats. Here, we describe the acoustic function and morphology of a unique stridulatory structure – the Ander's organ – in the relict orthopteran *Cyphoderris monstrosa* (Ensifera, Hagloidea). This species is one of just eight remaining members of the family Prophalangopsidae, a group with a fossil record of over 90 extinct species widespread during the Jurassic period. We reveal that the sound produced by this organ has the characteristics of a broadband ultrasonic anti-predator defence, with a peak frequency of 58±15.5 kHz and a bandwidth of 50 kHz (at 10 dB below peak). Evidence from sexual dimorphism, knowledge on hearing capabilities and assessment of local predators, suggests that the signal likely targets ground-dwelling predators. Additionally, we reveal a previously undescribed series of cavities underneath the organ that probably function as a mechanism for ultrasound amplification. Morphological structures homologous in both appearance and anatomical location to the Ander's organ are observed to varying degrees in 4 of the 7 other extant members of this family, with the remaining 3 yet to be assessed. Therefore, we suggest that such structures may either be more widely present in this ancient family than previously assumed, or have evolved to serve a key function in the long-term survival of these few species, allowing them to outlive their extinct counterparts.

## Abstract (precis)

**Highlighted Article:** Motion tracing, acoustic recording and micro-CT imaging reveal a unique ultrasonic organ in a relict insect. Statistical quantification of organ morphology reveals sexually driven loss of the organ in males.

## INTRODUCTION

Invertebrates have evolved a remarkable array of modes of communication, from chemical markers and aposematic colours to acoustic, vibrational and behavioural cues. Of these, some of the best studied are the mechanisms for conspecific communication, be it for kin recognition, competition or mate attraction (Drosopoulos and Claridge, 2006). But of all communication channels, the most crucial in the context of natural selection are the signals for anti-predator defence. These are typically divided into two classes: (1) primary defence mechanisms: the passive traits of an organism which operate regardless of predator presence; and (2) secondary defence mechanisms: those traits which function exclusively in direct or anticipated presence of a predator (Edmunds, 1974; Belwood, 1990). In animals as small as insects, passive physical defences such as spines may serve little resistance against vertebrate predators that are often much larger and faster, and have more sophisticated sensory and cognitive abilities (Montealegre-Z and Morris, 2004). As a consequence, insects have evolved a vast array of secondary defence mechanisms that fulfil an important role in avoiding predation (Belwood, 1990). These defences include the more characteristic traits of insects such as the hymenopteran sting, the spray of bombardier beetles (Eisner, 1958) and the saliva of assassin bugs (Edwards, 1961), and often function as a composite of behavioural, physical and chemical elements. The mechanisms by which these defences communicate to a predator differ greatly depending on both the predator and the prey. While the examples above highlight physical deterrents, many insects have evolved alternative ways to communicate to predators, such as visual displays or acoustics. Acoustic secondary defences have driven studies of predator–prey dynamics ever since early studies of invertebrate communication (Dumortier, 1963). In this context, sounds have been found to facilitate predator startle responses (Hoy et al., 1989), aposematic (Batesian) mimicry (Connor, 2014) and even signal jamming of echolocating predators (Connor, 2014).

In Ensifera (Insecta, Orthoptera; primarily bush-crickets or katydids, crickets, wētā and grigs), a great variety of primary and secondary anti-predator defences exist (Ter Hofstede et al., 2017). Their acoustic signals have evolved as a key mechanism in both conspecific communication and secondary anti-predator defence (Gwynne, 1995). These signals are typically produced by wing stridulation, in which a series of teeth (the file) on one wing engages with a scraper on the opposite wing to produce vibrations subsequently amplified as sound by specialized wing cells (Nielsen and Dreisig, 1970; Morris, 1999; Montealegre-Z, 2005). However, other mechanisms, including many which may have initially evolved as a by-product of a physical defensive component, are also observed (Masters, 1980, 1979; Field, 1993). Most well studied are the abdominal stridulatory mechanisms of Stenopelmatoidea (wētā, gryllacridids and allies; Field, 1993, 1982; Field and Glasgow, 2001; Field and Roberts, 2003). All known species of this superfamily exhibit femoro-abdominal stridulation, in which pegs or scrapers on the base of the hindlegs strike a series of abdominal teeth during defensive kicking behaviour (Field, 1982). Some also display tergo-tergal stridulation, in which successive abdominal tergites, possessing file and scraper mechanisms, strike one another during telescopic abdomen compression, resulting in sound production (Field, 1993). The sounds produced by these organs vary greatly in carrier frequency, from as low as 4 kHz, up to 32 kHz (Field, 1982; Heller, 1996). However, despite the ample knowledge of conspecific acoustic communication in Ensifera, the diversity and role of sound in the evolution of ensiferan anti-predator defence and descriptions of organ presence, remains limited (Field, 1982; Heller, 1996).

**Fig. 1.**
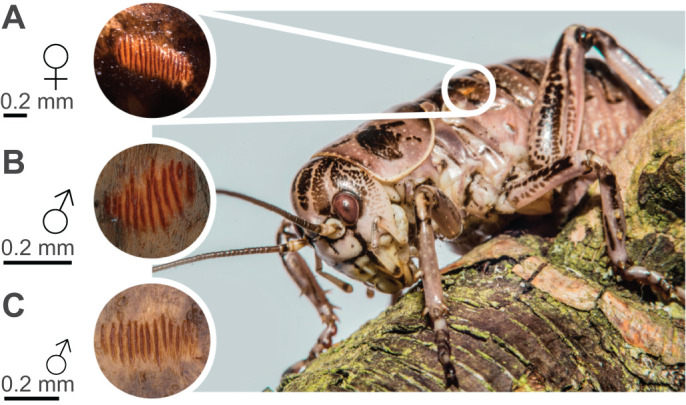
**Location of the Ander's organ of *Cyphoderris monstrosa* and examples of the stridulatory file.** (A) Adult female stridulatory file. (B) Adult male stridulatory file. (C) Juvenile male stridulatory file.

In the great grig, *Cyphoderris monstrosa* Uhler 1864, a relict ensiferan of the family Prophalangopsidae whose members were widespread during the Jurassic period (Ragge, 1955), secondary defence mechanisms appear to have taken on a variety of forms that differ between the sexes. When disturbed, males produce an acoustic signal by wing stridulation (the same mechanism as used for production of the conspecific song; Mason, 1996). However, all individuals of this species possess an additional stridulatory mechanism on the abdomen, the Ander's organ. In 1939, Swedish entomologist Kjell Ander hypothesised (reasoning from dead museum specimens) that this organ, a small pair of stridulatory files located on the lateral surfaces of the first abdominal tergite (Fig. 1), should function with an accompanying row of teeth along the posterior edge of the metanotum to produce sound (Ander, 1938). However, this hypothesis and any acoustic components of the organ have not since been investigated and the function or significance of the organ in the evolution of anti-predator defence remains unkown. *Cyphoderris monstrosa* offers a unique opportunity to understand ancient ensiferan biology; as one of only 8 extant species (Table S1) of a family containing over 90 species known only from fossils (Ragge, 1955; Cigliano et al., 2019; Gu et al., 2012). Therefore, 80 years on, we test Ander's hypotheses; statistically quantifying the morphology of the organ between sexes and life stages, investigating the acoustic signal, and describing the mechanism and function of the Ander's organ. In addition, we comment on the identification of similar morphological features across four of the other seven extant prophalangopsids and discuss the broader implications of these findings in the evolution of anti-predator acoustics.

## MATERIALS AND METHODS

### Live specimens

*Cyphoderris monstrosa* Uhler 1864 were hand-captured from William A. Switzer Provincial Park, Alberta, Canada (53°29′0.51″N, 117°49′32.55″W) after sunset at the Kelley's Bathtub day use area (*N*=57) as well as the area around the Blue Lake centre (*N*=53) between 6 and 13 July, 2019. From this sample of individuals collected as part of a project on temporal and geographic variation in selection, a subset [4 adult males, 4 adult females and 2 juveniles (1 of each sex)] were sent to the University of Lincoln, UK for bioacoustic experiments. Differences in sex were identified by sex-specific external genitalia.

While at the University of Lincoln, specimens were maintained on an *ad libitum* diet of bee pollen (Sevenhills, Wakefield, West Yorkshire, UK), fresh carrot, and cat biscuits (James Wellbeloved, Castle Cary, UK) and had access to water. Each animal was kept in an individual container in a cooled incubator (PHCbi MIR-154, PHC Holdings Corporation, Tokyo, Japan) on a 4-step temperature cycle (14 h:10 h light:dark cycle) around a mean of 8°C.

### Collection specimens

In addition to 4 of the individuals used in the bioacoustic study, a variety of other specimens were used for a study of the morphology of the Ander's organ in *C. monstrosa* (*N*=49) and homologous structures in congeners *Cyphoderris* *buckelli* (*N*=6) and *Cyphoderris* *strepitans* (*N*=4) and a closely related prophalangopsid species, *Paracyphoderris erebeus* (*N*=1). Details on collection locations and dates for all specimens are given in Table S2.

### Acoustic recordings

For measurements of the frequency composition and intensity of the Ander's organ acoustics, specimens were placed on a 30 cm^2^ surface of sound absorbent foam in an acoustic chamber. A B&K 1/8″ (3.2 mm) Type 4138 omnidirectional microphone (Brüel & Kjær, Nærum, Denmark), calibrated using a B&K Type 4237 sound pressure calibrator (Brüel & Kjær, Nærum, Denmark) was positioned 10 cm above the animal. The specimen was agitated using a soft paintbrush to test the best methods of inducing use of the Ander's organ. Brushing across the face elicited the most frequent response. The consequent acoustic signals were recorded using a PSV-500 internal data acquisition board (PCI-4451; National Instruments, Austin, TX, USA) via a preamplifier (B&K 2670, Brüel & Kjær, Nærum, Denmark) at a sampling frequency of 512 kHz. A high-pass filter set at 2 kHz was used to remove any low frequency background noise, and final recordings were saved as .txt files.

### Motion capture of Ander's organ mechanism

Specimens were immobilised prior to experiments by freezing at −2°C for 2 mins. This method was selected as a natural method of immobilisation, as the species has a freeze tolerance down to approximately −9°C (Toxopeus et al., 2016). While immobile, specimens were restrained with non-toxic Blu tack (Bostik Ltd, Stafford, UK) to a custom-made acrylic mount. Thin foam strips were attached laterally along the abdomen to prevent leg kicking but allow unrestricted abdomen movement. A 1 mm^2^ piece of reflective, non-scattering tape (Salzmann 3M Scotchlite Reflective Tape, Saint Paul, MN, USA) was then attached using non-toxic insect marking glue (E. H. Thorns Ltd, Wragby, UK) to the centre of the first abdominal tergite, which possesses the Ander's organ stridulatory files. Vertical motion of the abdomen could then be recorded from the reflection of this tape using a custom made opto-electronic photodiode motion detector (Montealegre-Z and Mason, 2005; Hedwig, 2000). The mounted animal was then left for 5 min to fully recover from freezing prior to recording. Data acquisition followed the same setup as with acoustic recordings, but with an additional recording channel for the motion detector (Fig. S1). All recordings were carried out at 18–22°C and 50% RH between 09:00 and 16:00 h.

### Signal analyses

Analysis of Ander's organ signals and averaging of frequency spectra was carried out using custom scripts written in MATLAB R2019a (MathWorks, Natick, USA). This analysis used 4096 FFT lines with signals recorded at a sampling frequency of 512 kHz. No filters were used on the signals other than a 2 kHz high-pass used during recording. Averaging consisted of 50 pulses from 6 bouts across 4 individuals. Signal waveforms and frequency spectra were also plotted using MATLAB.

### Morphological analysis

In adult and juvenile males and females used in the bioacoustic study, following death, the length and width of the Ander's organ stridulatory file, as well as tooth distribution and density, was imaged using an Alicona InfiniteFocus microscope (Bruker Alicona Imaging, Graz, Austria) at 10× objective magnification, resulting in images with a resolution of ∼100 nm. Length, distribution and density of teeth were defined and measured using standardized techniques (Fig. S2). Specimens from collections prior to 2019 (Table S2) had their Ander's organs (left and right) imaged using a Leica Si9 stereomicroscope (Leica Microsystems, Wetzlar, Germany). Morphological dimensions of the Ander's organ were measured from microscope photographs using ImageJ (Schneider et al., 2012) by standard scale bar calibration, following the same standardized measurement definitions. Microscope photographs of head width and left femur length were bundled using tpsUtil v1.70, and tpsDig2 v2.26 (http://sbmorphometrics.org/) was used to place landmarks from which linear dimensions were calculated.

### μ-CT imaging and measurement

X-ray µ-CT of one adult of each sex of was performed using a SkyScan 1172 µ-CT scanner (Bruker Corporation, Billerica, MA, USA) with a resolution of 3 µm (45 kV source voltage, 185 µA source current, 400 ms exposure and 0.1 deg rotation steps). Prior to the scan, specimens (already preserved in ethanol) were removed from their preservation containers and positioned in a custom-built holder in the CT scanner. µ-CT projection images were reconstructed to produce a series of orthogonal slices with NRecon (v.1.6.9.18, Bruker Corporation, Billerica, MA, USA), and the 3D image captured using CTvox (Bruker Corporation, Billerica, MA, USA). 3D segmentation and rendering of the organ, and measurements of sub-organ cavity length, was carried out in Amira-Aviso 6.7 (Thermo Fisher Scientific, Waltham, Massachusetts, USA). We used the length measurements of the cavities to calculate an estimate of resonance. This was calculated by the assumption that the cavity acts as a cylindrical tube. In such tubes, the fundamental frequency (*f*_0_) corresponds to the wavelength that is twice the length of the tube, calculated as:(1)
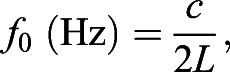
where *c* is the speed of sound in air, and *L* is the length of the tube in metres.

### Statistical analyses of Ander's organ morphology

We used seven measurements to describe Ander's organ morphology in *C. monstrosa*: (1) number of stridulatory file teeth; (2) average file tooth length; (3) standard deviation of file tooth length; (4) mean inter-tooth distance; (5) standard deviation of inter-tooth distance; (6) metanotal spine number; and (7) file length. Measurements 3 and 5 allowed for a quantification of variation in organ morphology within individuals. In all cases, we averaged the calculated values for both left and right Ander's organs of each individual prior to statistical analysis. We analysed variation in Ander's organ morphology using a multivariate general linear model (GLM) with Sex (male or female), Stage (juvenile or adult) and their interaction as fixed factors, and the above seven measurements as dependent variables. All statistical analyses were completed in IBM SPSS Statistics v.26 (IBM Corporation, 2019).

## RESULTS

### Morphology of the Ander's organ

**Fig. 2.**
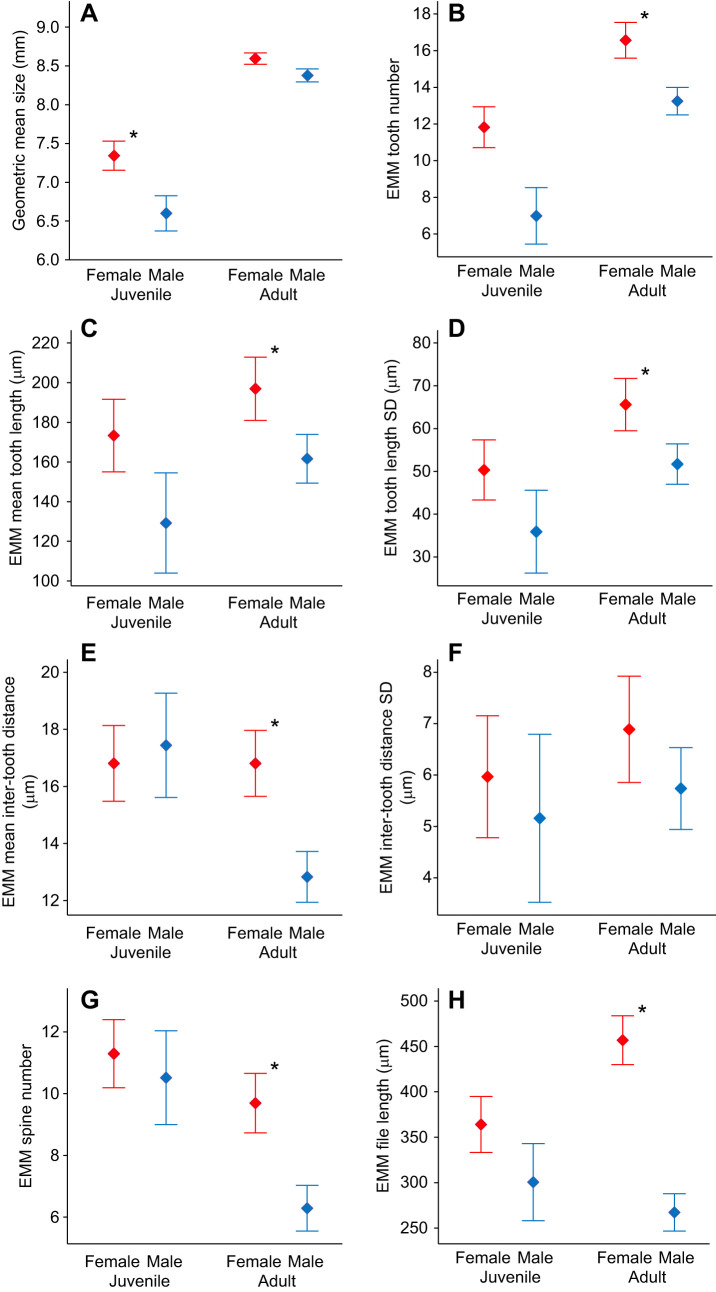
**Differences in body size and estimated marginal means of Ander's organ morphology in *C. monstrosa* based on sex and life stage.** (A) Geometric mean size (GMS) is calculated as the square root of head width×left femur length. (B–H) Estimated marginal means (EMMs) for indicated factors. Error bars represent 1 s.e.m. Asterisks indicate statistically significant differences at *P*<0.05. Statistical comparisons of Ander's organ measurements (B–H) are shown for adults and juveniles separately because of a Sex×Stage interaction effect (see Results).

The Ander's organ of *C. monstrosa* is present in both sexes, and all assessed life stages (Fig. 1), with larger individuals possessing larger organs (Table 1, Fig. 2). Given that body size differs both between females and males and between adults and juveniles (Table 1, Fig. 2A), we included body size as a covariate in our analysis of sex differences and developmental changes in Ander's organ morphology. We ran a multivariate GLM with seven Ander's organ measurements as dependent variables (see Materials and Methods), initially including all possible three- and two-way interaction terms between the fixed factors (Sex and Stage) and covariate (Size). All nonsignificant interactions were removed in a stepwise fashion starting with the three-way interaction (Sex×Stage×Size: *F*=1.164, d.f.=7, 35, *P*=0.348), then the Sex×Size interaction (*F*=0.800, d.f.=7, 36, *P*=0.592), and then the Stage×Size interaction (*F*=1.972, d.f.=7, 37, *P*=0.086). Ander's organ morphology is sexually dimorphic, but this dimorphism differs when considering either adults or juveniles (Sex×Stage interaction, *F*=2.567, d.f.=7, 38, *P*=0.029). We therefore ran separate multivariate GLMs for adults and juveniles with Sex as the fixed factor, Size as the covariate, and their two-way interaction as independent variables. In neither adults nor juveniles was the Sex×Size interaction term statistically significant (adults: *F*=0.511, d.f.=7, 22, *P*=0.816; juveniles: *F*=2.049, d.f.=7, 7, *P*=0.182) and so it was removed from both models. After controlling for body size, female *C. monstrosa* had larger Ander's organs than males did, but that difference was only statistically significant for adults (adults: Sex *F*=14.399, d.f.=7, 23, *P*<0.001; juveniles: Sex *F*=1.601, d.f.=7, 8, *P*=0.261). This multivariate pattern was replicated for each individual measurement (Fig. 2), with females having more stridulatory teeth, longer teeth, greater tooth length standard deviation, longer inter-tooth distance, greater inter-tooth distance standard deviation, more spines and longer organs than males, but this was only statistically significant in adults (Fig. 2).

**Table 1.**
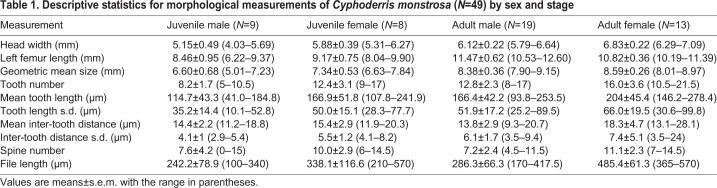
**Descriptive statistics for morphological measurements of *Cyphoderris monstrosa* (*N*=49) by sex and stage**

An assessment of other extant prophalangopsid species (*C. buckelli*, *C. strepitans*, *Paracyphoderris erebeus*) uncovered undescribed morphological structures with varying levels of similarity to the Ander's organ, sharing the presence of trailing spines or hairs along the posterior edge of the metanotum (Fig. S3). In *Paracyphoderris erebeus*, an Ander's-like organ was found, with ridges acting as stridulatory teeth, and 8 clear metanotal spines (*N*=1, Fig. S3A). Owing to the low number of accessible specimens for these additional species, a detailed quantification and comparison of morphological parameters has not yet been possible.

### Acoustic signal analysis

We attempted to elicit use of the Ander's organ in 4 adult males, 4 adult females and 2 juveniles (1 of each sex). Adult males consistently failed to produce a sound using the organ, instead producing an acoustic defence with tegmina when perturbed (Fig. S4). All adult females and all juveniles successfully produced sound using the organ on at least one occasion, and of these, all but 1 adult female provided acoustic recordings suitable for temporal and spectral analysis.

**Fig. 3.**
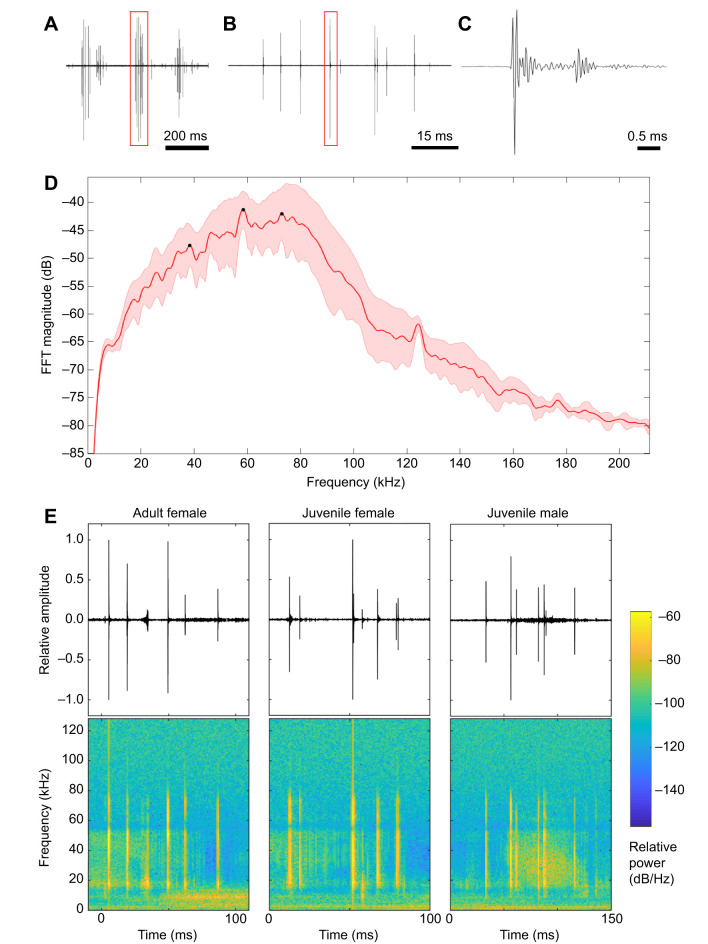
**Acoustic analysis of Ander's organ signals.** (A) Waveform of multiple Ander's signals, with signal shown in B highlighted in red. (B) One bout, with signal shown in C highlighted in red. (C) An individual pulse of one bout. (D) Relative magnitude mean frequency spectrum over individual pulses (*N*=50; the solid red line signifies the mean, the shaded area ±1 s.d.). (E) Example signal waveforms (top) with spectrograms (bottom) from an adult female, juvenile female and juvenile male.

The signal generated by the Ander's organ of female and juvenile *C. monstrosa* was found to consist of temporally unstructured pulse sequences (or bouts; Fig. 3A–C), each containing a series of broadband ultrasonic pulses, with a peak frequency of 58±15.5 kHz (mean±s.d., *N*=50 pulses from 5 animals; Fig. 3D). The waveform of the signal is highly variable in amplitude, ranging from ∼30 to 100 dB SPL (re. 20 µPa at 1 cm). Pulses also display a high, but inconsistent, repetition rate at 69.1±22.3 pulses/second, with 4-12 pulses per bout (Fig. 3B). Each pulse is extremely rapid, with an average duration of 0.30±0.02 ms (Fig. 3C). Welch's power spectral density (PSD) analysis revealed the signal is highly broadband, with a bandwidth ranging from 40 to 90 kHz at 10 dB below the maximum energy peak (Fig. 3D).

### Ander's organ mechanism and additional defensive components

**Fig. 4.**
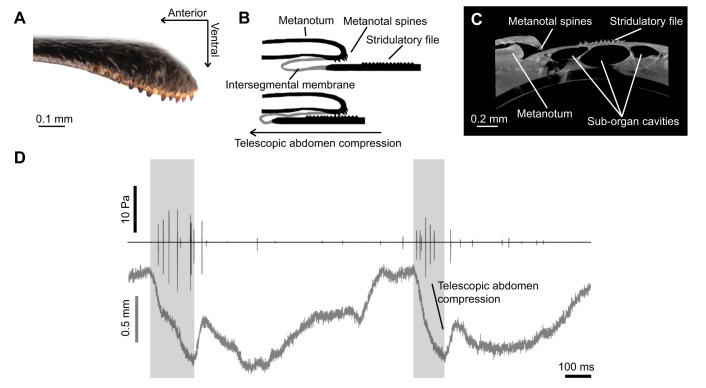
**The mechanism for Ander's signal generation.** (A) Metanotum edge with spines that scrape the abdominal file. (B) Mechanism of Ander's signal generation by telescopic abdomen compression. (C) Cross-section of the Ander's organ, showing the presence of air-filled cavities beneath the stridulatory file. (D) Motion of Ander's mechanism, showing the relationship between the motion of the abdomen (bottom) and the consequent acoustic signal (top). Highlighted areas show full signals, which occur exclusively during telescopic abdomen compression.

**Fig. 5.**
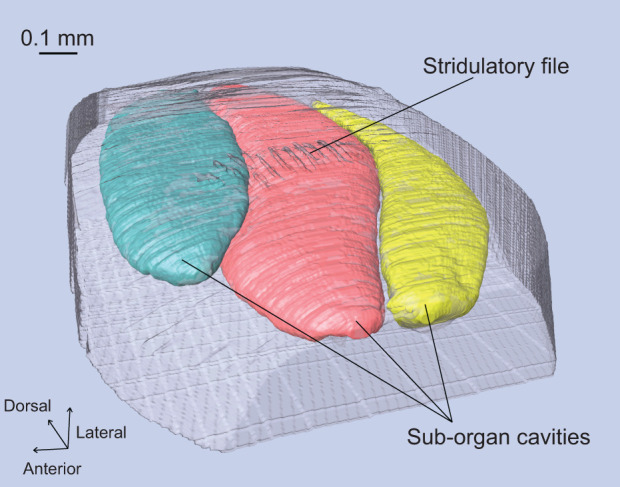
**3D reconstructed morphology of the Ander's organ of an adult female highlighting the anatomy of the sub-organ cavities.**

We attached a 1 mm^2^ piece of reflective tape to the metanotum of partially restrained specimens of *C. monstrosa* and tracked Ander's organ use by following the movement of the tape with a custom made opto-electronic photodiode motion detector (Montealegre-Z and Mason, 2005; Hedwig, 2000). This was coupled with a microphone to record the association between motion and acoustic signal (for full details, see the Materials and Methods). Motion traces confirmed the mechanism of Ander's organ is tergo-tergal stridulation, in which the first abdominal tergite, containing the stridulatory files, moves by telescopic abdomen compression underneath the posterior edge of the metanotum which possesses several spines that act as scrapers (Fig. 4B). The acoustic signals are generated during this abdomen compression, but low amplitude signals are also generated as the file disengages the metanotum during abdomen expansion (Fig. 4D). Organ use was exclusively exhibited by females and juveniles during physical contact, and was accompanied by either leg kicking behaviour while the animal was on its back, mandible opening, or both, forming a composite signal. This sound may also be amplified by a trio of tracheal cavities that underlie the stridulatory file (Fig. 4C and Fig. 5). These cavities are independent of the respiratory tracheae and were present in all assessed individuals (1 adult male and 2 adult females), but absent under other abdominal tergites, suggesting they are a specialised structure for amplification. We used the actual length measurements of the cavities to provide a preliminary estimate of the resonant frequency of each cavity in an example adult female, to discern whether these structures are likely to be involved in signal amplification. The 3 cavities had lengths of 1850.6 µm, 2646.9 µm and 1969.3 µm, which, using Eqn 1, suggest a resonance of 92 kHz, 64 kHz and 87 kHz, respectively.

## DISCUSSION

In his initial observations of this ‘chirping organ’, Ander provided no comprehensive explanation of the function of the stridulatory mechanism (Ander, 1938) and the organ as such. Various hypotheses were made from his morphological description which we here investigated; namely that sounds are generated during organ use and proving that the contraction of the abdomen forms the more significant motion in acoustic signal generation. The broad frequency range of the acoustic signal is almost certainly facilitated by extreme irregularity in organ morphology and feature distribution, highlighted by the variation in tooth length and inter-tooth distance within individuals (Fig. 2D,F; Fig. 4C,E). In addition, the finding of a cavity underneath the organ suggests a resonant mechanism for amplification of the sound. This broad frequency range, the impulsive waveform of the sound and organ use only during physical contact are key indicators that the organ functions as an acoustic anti-predator defence (Masters, 1980, 1979; Haskell, 1974; Bauer, 1976), and thus we support this hypothesised function of the signal. This is further supported by the additional observed behaviours associated with organ use; namely leg kicking and mandible opening, which could serve to physically deter a predator. The amplitude and frequency spectrum of the signal leads to the conclusion that *C. monstrosa* is targeting predators with an ultrasonic hearing range that extends to at least 60 kHz, most likely to elicit an acoustic startle response to increase chances of survival (Sillar et al., 2016). The assessment of sub-organ cavity resonance suggests that the measured organ can, in theory, produce broadband resonant sound amplification from ∼64 kHz to 92 kHz. As individuals possess one organ per body side which could display an increased morphological variation and consequent resonance, we believe it is plausible that amplification is the role of these cavities; however, a more robust modelling, such as by finite element modelling, will prove useful in confirming this hypothesis.

Quantification of the morphology of the organ and an observed lack of Ander's organ use in males also support the notion of functional loss in males posed by Ander (1938), with females displaying larger organs in every measured parameter. This may initially suggest a difference in predation risk between groups, perhaps as a result of behavioural ecology. During the mating season, males climb several metres into the canopy, ascending higher as the evening progresses. Females are assumed to spend their days in underground burrows, only ascending trees at night to locate singing males (Morris and Gwynne, 1978; Mason, 1996). However, both sexes have been observed on/under the ground, and there are as yet no full descriptions of the disparity in male and female daily activities.

A more plausible explanation is that such a dimorphism represents an example of sexual selection. The sex by stage interaction of morphological analysis indicated that males have significantly smaller organs, but only when they are sexually mature, suggesting that features exclusive to adult males are prioritized over those for disrupting a predator, and the pre-existing antipredator signal (Ander's organ) has lost its function as a result. We identify these sexually selected features as the tegminal calling song for mate attraction, and the development of thick hindwings as a nuptial gift for female consumption (Judge et al., 2011). These likely render the Ander's organ useless, as both pairs of wings overlie the stridulatory file and metanotum.

Additionally, while it could be argued that males may not require the functional organ as they can produce a warning signal by tegminal stridulation (Morris and Gwynne, 1978), this sound does not have the same broadband energy or ultrasonic components as the Ander's organ signal (Fig. S4), so may be used against a different predator, or one whose hearing range includes both the male tegminal aggression, and the Ander's organ ultrasonics. Females may indeed be at higher predation risk than males during their time spent following the male calls for mating, a process known as phonotaxis, which would prolong predator exposure. In addition, juveniles are likely under strong selection to acquire food to grow and mature, and females under fecundity selection to acquire sufficient nutritional resources to produce embryos. Previous studies of grigs have shown that nutritionally-deprived adult females are more likely to mate and feed on male hind wings under close conditions in the lab (Judge et al., 2011; Johnson et al., 1999), possibly indicating that they may engage in more phonotaxis when hungry, further increasing predator exposure. On the other hand, adult males feed little in captivity (K.A.J., pers. obs.) and may also not feed much in the wild if they acquire all the resources needed to mate before moulting to adulthood. Overall, this difference supports the concept that females and juveniles have an increased likelihood of predator exposure. A female bias in morphological disparity may suggest an element of maternal–offspring communication; however, the frequency composition of the signal far exceeds the hearing range of this species (Mason, 1991).

There are several hypotheses of how an acoustic signal such as that of the Ander's organ could be used to evade predation. The simplest of these is the predator startle hypothesis, by which the impulsive stop/start waveform of the sound at high sound pressure levels acts to frighten a predator, increasing the temporal window for escaping death (release call) (Masters, 1979). These signals may also have evolved as a form of Batesian mimicry for nocturnal species (Masters, 1979; Heller, 1996), whereby insects without additional secondary defences (unarmed) have evolved the same acoustic signal as insects with additional defences. This allows unarmed species to take advantage of the pre-programmed predator association between the sound and the true defence, and deter the same predator with a false warning. Such unarmed insects tend to represent the greatest presence of stridulatory warning signals (Heller, 1996), suggesting this mode of mimicry (Masters, 1980, 1979; Heller, 1996). Others have posed that due to similar spectral and temporal characteristics, and the possession of additional defensive components in almost all insects, these disturbance sounds exhibit a widespread Müllerian mimicry (Masters, 1980), whereby the signals always represent a true warning to the predator, with no unarmed species. This mimicry has been confirmed in the acoustic behaviour of deaf moths (O'Reilly et al., 2019) and for ultrasonic signals, may disrupt echolocating predators (Fullard et al., 1979; Conner and Corcoran, 2012). In *C. monstrosa*, the signal in this context may serve as a warning of the potential bite the predator would receive if it were to prolong its attack.

Although the obvious predators that come to mind when considering ultrasonic capabilities are bats, the auditory processing of *C. monstrosa* provides evidence for non-bat predation, as this species possesses one population of auditory receptors tuned to low frequencies, and a second type of auditory receptor tuned to a broader frequency range that includes the male song (Mason et al., 1999). As frequency discrimination beyond these two categories of receptor is not possible, *C. monstrosa* is unlikely to use its high frequency receptors for predator detection (Mason et al., 1999; Wyttenbach et al., 1996), as these receptors are preoccupied with the role of detection of the male song. Instead, it is the other population of receptors, best tuned to low frequencies ∼2 kHz, that are suspected to be associated with detecting terrestrial predators moving through a substrate (Mason et al., 1999). However, this does not mean that *C. monstrosa* is unable to detect surface gleaning bats, as it is known from other orthopteran insects that cercal organs may also be involved in the detection of aerial predators, via the motion of hairs in response to wind produced by the wings of an approaching bat (Hartbauer et al., 2010). *C. monstrosa* possesses the necessary cerci, and an investigation into their function could prove beneficial to investigating this alternate mode of predator detection.

Several bats do indeed share a geographic overlap with *C. monstrosa*. These species are the big brown bat (*Eptesicus fuscus*), the silver-haired bat (*Lasionycteris noctivagans*) and the hoary bat (*Lasiurus cinereus*; Naughton, 2012). However, these bats are primarily aerial predators, hunting on the wing, and *C. monstrosa* is not capable of flight. *E. fuscus* for example is a common species across the habitat range of *C. monstrosa*, but is considered to be a ‘beetle-specialist’, with nearly 90% of its diet consisting of small to mid-sized flying insects (Naughton, 2012; Surlykke et al., 2009; Clare et al., 2014). *Lasiurus cinereus* is also adapted for fast, unmanoeuvrable flight, and so mainly feeds on small flying insects (Barclay, 1985, 1986). *Lasionycteris noctivagans* flies more slowly and manoeuvrably than *L. cinereus* (Conner and Corcoran, 2012), often feeding in clearings over relatively short distances (Barclay, 1985, 1986), so is a potential surface-gleaning predator. Recorded diets of these predators also often contain high levels of unidentified insect matter (up to 15%; Barclay, 1985, 1986). This could include *C. monstrosa* in the category of generally large, unknown ground-dwelling insects, but as a result of the argument raised above and the fact that a large percentage of their diet consists of small flying insects (Barclay, 1985, 1986), we believe this to be unlikely. Owing to this lack of evidence for bat predation, we suggest echolocating and high frequency communicating shrews as a more likely ultrasonic predator. The two species that coexist with *C. monstrosa* are the cinereous shrew (*Sorex cinereus*) and dusky shrew (*Sorex monticolus*). *Sorex cinereus* has been reported to echolocate, particularly in novel environments and while foraging (Gould, 1969). While the echolocation bandwidths of these particular two species are unknown, the known upper frequencies of other shrew species vary from ∼30 to 95 kHz (Gould, 1969; Sales and Pye, 1974; Forsman and Malmquist, 1988; Thomas and Jalili, 2004), meaning they very likely have the capability to hear the frequency range of the Ander's organ signal.

There are also certain non-echolocating species which are insectivorous and share a geographic overlap. The northern flying squirrel (*Glaucomys sabrinus*), may be an interesting candidate as one of the only species likely to encounter *C. monstrosa* both in the trees and on the ground. However, invertebrates make up less than 1% of the diet of this species (Maser et al., 1985). Other species include the yellow-pine chipmunk (*Tamias amoenus*), red squirrel (*Tamiasciurus hudsonicus*), western jumping mouse (*Zapus princeps*), long-tailed vole (*Microtus longicaudus*) and deer mouse (*Peromyscus maniculatus*; Naughton, 2012). Mice have generally been found to produce communication signals up to ∼110 to 120 kHz (Portfors, 2007; Kalcounis-Rueppell et al., 2006), so may also represent a key predator; particularly *P. maniculatus*, which is likely abundant within the range of *C. monstrosa* and lives a heavily arboreal lifestyle, allowing for predation of *C. monstrosa* both on the ground, and in the trees. While this species is known to commonly eat insects (Whitaker, 1966), there has not yet been a study of the diet of *P. maniculatus* within this region.

We suspect the predation ecology of *C. monstrosa* to be similar to the New Zealand wētā (Anostostomatidae), which possess similar stridulatory organs, and are an integral part of the diet of stoats and other ground-dwelling mammals (Field, 1993; Smith et al., 2005). However the only known native predator of wētā capable of hearing ultrasound is the (ground-hunting) lesser short-tailed bat (*Mystacina tuberculate*; Parsons, 2001), and so increased knowledge on the ultrasonic hearing capabilities of the other predators of wētā could assist in refining this comparison.

In another species closely related to *C. monstrosa*, *Paracyphoderris erebeus*, the morphology of a structure with similarity to the Ander's organ (Fig. S3) suggests the ability to produce a similar signal. We propose, based on the morphology of both the Ander's organ and the tergal stridulatory mechanisms of certain wētā (*Deinacrida* spp.; Field and Roberts, 2003), that it has a similar function. Remnants of these structures and others in 5 of the 8 known extant prophalangopsid species suggest that abdominal stridulation could be a common trait of the family (Fig. S3). Alternatively, it may be that this structure is a key convergent adaptation that, along with other traits, has allowed these species to persist where other members of the family have gone extinct.

We believe that the discovery of ultrasonic signals in *C. monstrosa*, and the presence of these morphological traits across other extant prophalangopsids, provide a foundation of evidence that ultrasonic stridulatory organs could be present earlier in the history of Ensifera than previously assumed. Observations of the natural behaviour of *C. monstrosa* and its predators would provide more information on the function of the Ander's organ. In addition, efforts should be made to discover such organs in fossil species, as this may provide insight into the origin of ultrasonic anti-predator defences, and the hearing ranges of ancient terrestrial predators.
